# X-ray attenuation of bone, soft and adipose tissue in CT from 70 to 140 kV and comparison with 3D printable additive manufacturing materials

**DOI:** 10.1038/s41598-022-18741-4

**Published:** 2022-08-26

**Authors:** Xiangjie Ma, Michael Figl, Ewald Unger, Martin Buschmann, Peter Homolka

**Affiliations:** 1 Center for Medical Physics and Biomedical Engineering, Medical University of Vienna, Vienna, Austria; 2 Division of Medical Radiation Physics, Department of Radiation Oncology, Medical University of Vienna, Vienna, Austria; 3 Present Address: Key Laboratory of Radiological Protection and Nuclear Emergency, National Institute for Radiological Protection, Chinese Center for Disease Control and Prevention, Beijing, China

**Keywords:** Applied physics, Medical imaging, Radiography, Tomography

## Abstract

Additive manufacturing and 3D printing are widely used in medical imaging to produce phantoms for image quality optimization, imaging protocol definition, comparison of image quality between different imaging systems, dosimetry, and quality control. Anthropomorphic phantoms mimic tissues and contrasts in real patients with regard to X-ray attenuation, as well as dependence on X-ray spectra. If used with different X-ray energies, or to optimize the spectrum for a certain procedure, the energy dependence of the attenuation must replicate the corresponding energy dependence of the tissues mimicked, or at least be similar. In the latter case the materials’ Hounsfield values need to be known exactly to allow to correct contrast and contrast to noise ratios accordingly for different beam energies. Fresh bovine and porcine tissues including soft and adipose tissues, and hard tissues from soft spongious bone to cortical bone were scanned at different energies, and reference values of attenuation in Hounsfield units (HU) determined. Mathematical model equations describing CT number dependence on kV for bones of arbitrary density, and for adipose tissues are derived. These data can be used to select appropriate phantom constituents, compare CT values with arbitrary phantom materials, and calculate correction factors for phantoms consisting of materials with an energy dependence different to the tissues. Using data on a wide number of additive manufacturing and 3D printing materials, CT numbers and their energy dependence were compared to those of the tissues. Two commercially available printing filaments containing calcium carbonate powder imitate bone tissues with high accuracy at all kV values. Average adipose tissue can be duplicated by several off-the-shelf printing polymers. Since suitable printing materials typically exhibit a too high density for the desired attenuation of especially soft tissues, controlled density reduction by underfilling might improve tissue equivalence.

## Introduction

Materials mimicking body tissues in a phantom, defined as a model of the human body or body part with respect to the features or interactions of interest, are used in widespread applications in clinical simulation and biomedical research^1^. Phantoms are applied in all diagnostic and therapeutic radiologic modalities, like medical ultrasound imaging, MRI, nuclear medicine and CT^2^, mammography^3,4^, projective imaging and interventional radiology, and radiation therapy^5^. In X-ray imaging applications, these phantoms “simulate the modification of the radiation field caused by absorption and scattering in the body tissues or organs of interest”^6^.

Additive manufacturing using 3D printing as manufacturing method represents a flexible and cost-efficient way to create phantoms, offering a large degree of flexibility in the design^2,7,8^. This technology is particularly useful, since it allows a realistic and accurate representation of the anatomy and the properties of tissues^4^, structures and internal textures^9–15^. Additive manufacturing and 3D printing paved the way for the transition from simple test objects and phantoms, such as geometric and slab phantoms, to anthropomorphic or at least semi-anthropomorphic phantoms. This higher degree of fidelity supports the transition from testing and optimizing *technical* image quality parameters to assessing *diagnostic* image quality^9^.

Phantoms utilizing the design and manufacturing potentials of additive manufacturing and 3D printing have been developed for a wide range of applications in medical physics, including dose determination^5,16^, quality control, system characterization, image quality measurement and optimization^10,14,17^, or teaching purposes. Other applications where the ability of 3D printing to manufacture phantoms with accurate individual and patient specific features is exploited include operative rehearsal and visualization of complex surgical procedures^18,19^. The requirements on the materials used in these phantoms differ depending on the intended use and modality. For the latter, e.g., the haptics of the printing materials is most important, while in most imaging applications we are interested in phantoms resulting in tissue equivalent image signals and contrasts.

However, most well-established tissue mimicking materials cannot be processed with 3D printers, resulting in a need to develop 3D printable materials mimicking imaging or dosimetric properties of body tissues^20^, and to catalog available materials according to their radiographic characteristics^21,22^. Development of materials and printing methods used for phantoms include doping printing filaments and resins with additives like materials with higher atomic numbers as, e.g., bismuth, tungsten or iodine^23,24^, or radioactive tracers^25–27^, but also aims at adjusting mass density of the printouts by adapting the printing process^22,28,29^.

Phantoms materials used for X-ray imaging applications need to be well characterized in terms of their interaction properties with the X-ray photons, most importantly X-ray absorption and inelastic scattering. As stated by the ICRU definition of radiographic phantoms^6^, the modification of the radiation field by the phantoms needs to cover both, primary and secondary radiation fields. Materials used in phantoms need to mimic interaction properties of the tissues or materials they substitute as closely as possible, at least in the photon energy range they are intended to be used. In the keV energy range, this is especially complicated by the contribution of the photoelectric effect with its strong energy and atomic number dependence. Thermoplastic polymers used in 3D printing with fused deposition modeling (FDM) or selective laser sintering (SLS), as well as photo cured resins used in polyjet printers, stereolithography (SLA), and digital light processing (DLP) systems typically have a too low effective atomic number compared to water or soft body tissue. This results in a poor replication of the energy dependence of the X-ray interaction of tissues, often limiting their use to a rather narrow photon energy range. Craft et al.^30^ state, that currently no commercially available materials can exactly replicate bone or lung tissue, indicating that the choice of materials available for X-ray phantoms is still limited. However, mimicking soft tissue over a wide energy range with a single material for keV X-ray photons is more difficult than duplicating X-ray properties of bone due to the chemical composition of the thermoplastics and photo curable resins^21^. In bone tissues, including soft spongious to hard cortical bone, not only the total attenuation of bone tissues is strongly dependent on the bone mineral content, but also the energy dependence. Since the chemical composition—and thus the average effective atomic number—in dense bone is very different to soft bone with bone marrow, the full range of bone densities needs to be considered.

X-ray attenuation of common additive manufacturing and 3D printing materials have been measured by several groups. Especially in FDM, printing parameters can also strongly influence X-ray attenuation properties^31^. Therefore, great care must be taken to obtain compact print samples avoiding printing artefacts resulting in uncontrolled underfilling causing too low density, and thus X-ray attenuation, due to microscopic or macroscopic voids often inherent to the printing technologies. This is especially an issue in FDM and SLS printers using thermoplastic polymers. As a result, X-ray attenuation properties like CT numbers differ greatly between publications for the same 3D printing materials^21^. In order to improve transferability and reproducibility of the results, the printing parameters of the most important polymers were systematically optimized in a previous study^21^. By adjusting the printing parameters for FDM polymers, the highest achievable printout density, ideally equal to the mass density of the filament, was determined systematically. Five thermoplastic polymers could be printed with a mass density exactly resembling the filament density (± 0.00 g/cm^3^), and eleven materials with a deviation of 0.01 g/cm^3^ indicating near optimum achievable density. The samples were then scanned in CT in a water phantom applying all tube potentials available in order to obtain reference values for their X-ray attenuation.

The main focus of this work was to experimentally determine typical representative values including their energy dependence of CT numbers of fresh tissue for tube potentials between 70 and 140 kV. This is intended to serve as reference for phantom materials, and to compare these to appropriate additive manufacturing materials, and to assist material selection in printed phantoms for best tissue equivalence.

## Results

### Whole bone and cortical bone

CT numbers in Hounsfield Units were determined for total bone comprising of whole bone samples for bovine rib bone and a porcine tibial and fibular head. In these data, reflecting total average bone, all bone densities present in the sample are averaged.

Cortical bone samples were measured in bovine rib bone, and the porcine tibial and fibular shaft.

**Figure 1:**
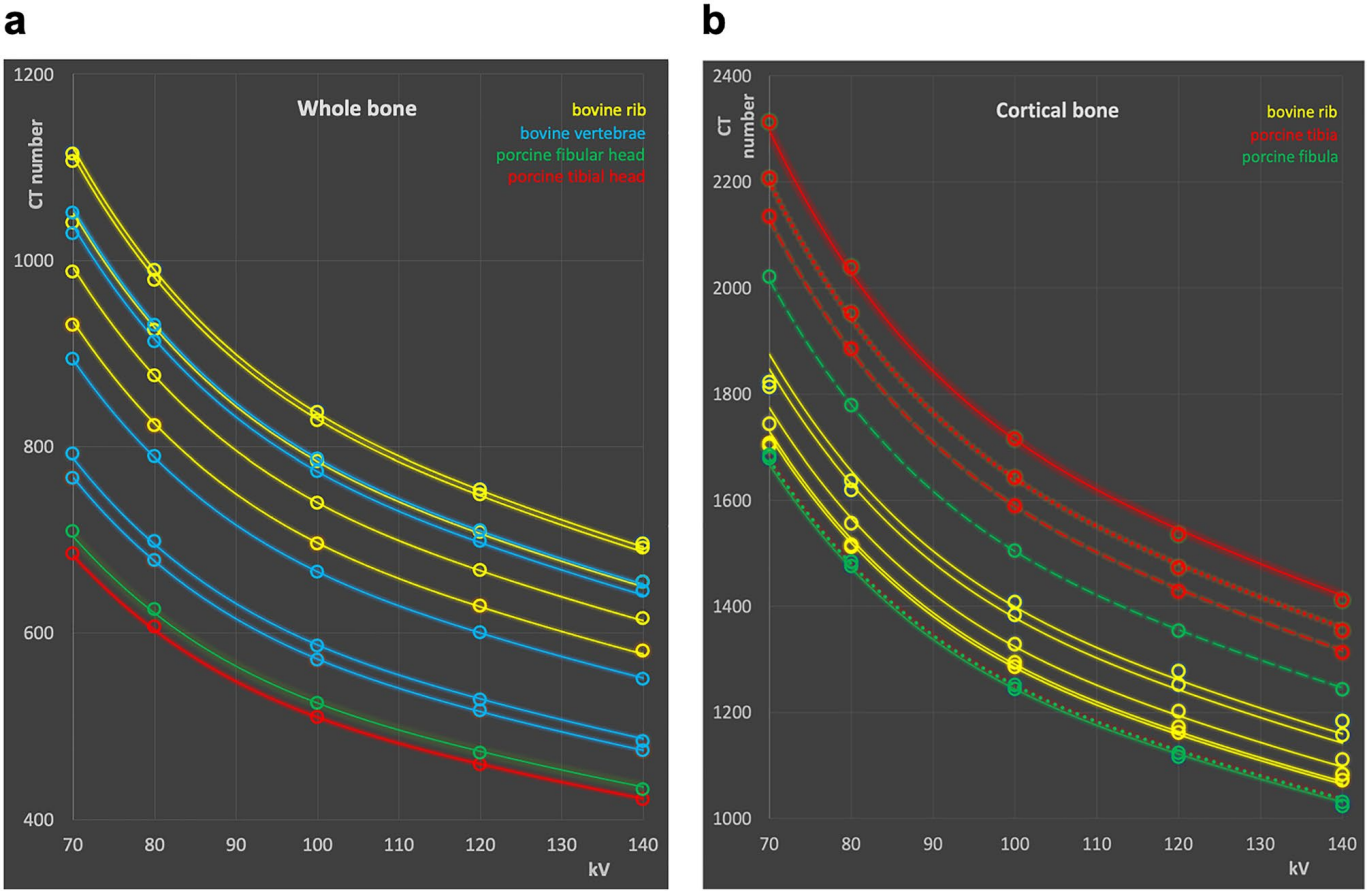
CT numbers measured in the bone samples. (**a**) Whole and (**b**) cortical bone. Circles indicate measured CT values; lines correspond to model equations. Broken lines in (**b**) indicate measurements in the distal porcine tibia or fibula, solid lines in the mesial part, and dotted line measurement over the whole bone shaft, respectively.

Hounsfield numbers from 70 to 140 kV measured in total bone, and cortical bone are shown in Fig. 1a and b, respectively, for the bovine and porcine bone samples. Multiple measurement curves shown for bovine rib bone correspond to values measured on different ribs. At 120 kV used as reference, the whole bone samples exhibited CT numbers from 458 to 754 HU. These corresponded to a range of 685–1114 HU at 70 kV, and 421–695 HU at 140 kV, respectively.

Cortical bone samples covered a range from 1116 HU (mesial porcine fibula) to 1536 (mesial porcine tibia) at 120 kV. This corresponded to 1679–2313 HU at 70 kV, and 1024–1411 HU at 140 kV.

### CT numbers of segmented bone ROIs

**Figure 2:**
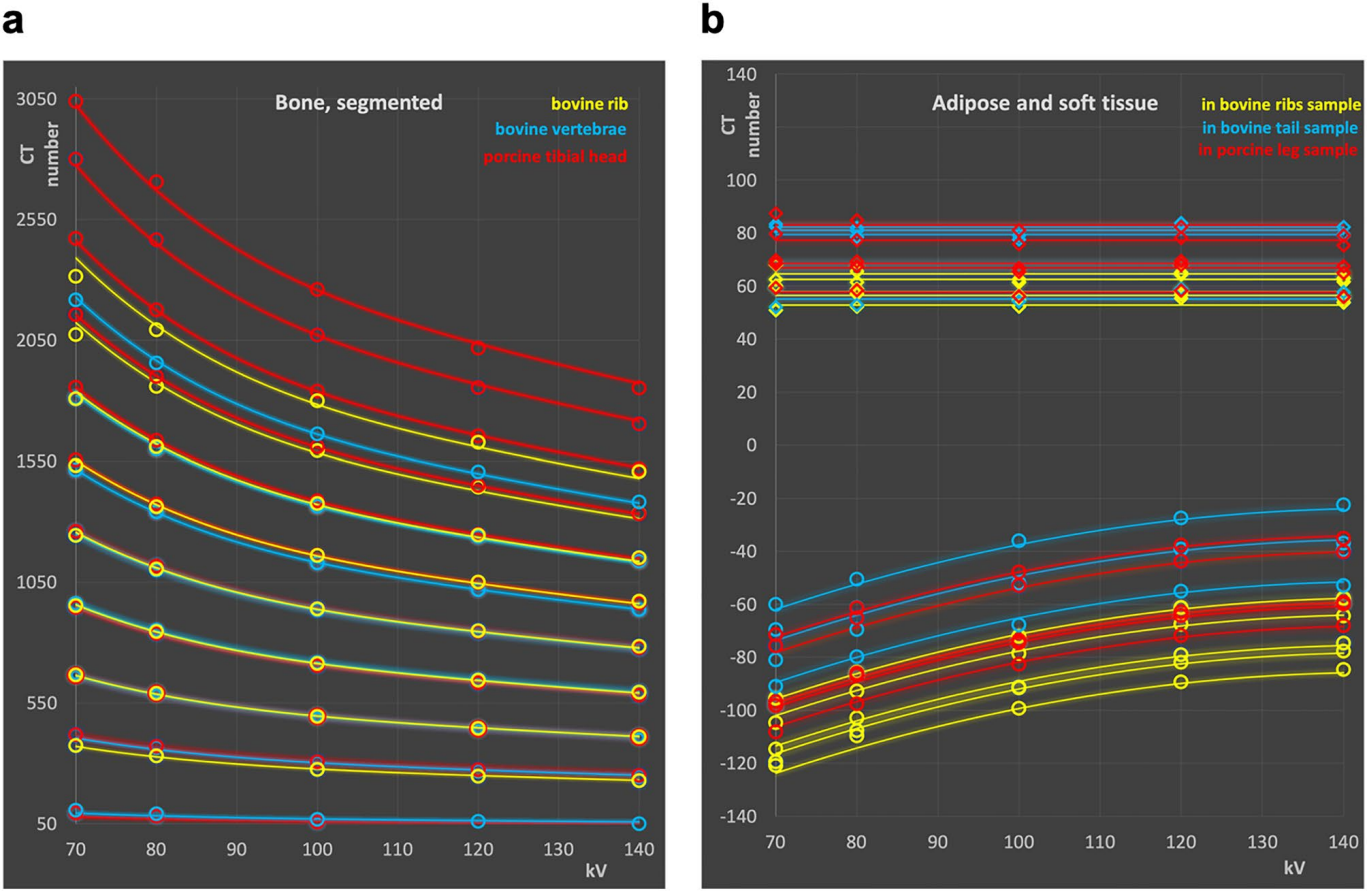
CT numbers from segmented bone samples (**a**), and soft tissues (**b**). Diamonds represent soft tissue (mainly muscular tissue), and circles adipose tissue. (**a**) and (**b**): Symbols: measured points, lines: model equation.

In addition to measuring total bone and cortical bone, the bovine rib bone samples, bovine vertebrae and the porcine tibial head were segmented into areas of differently hard bone, and their CT values determined. The segmentation of the bone in volumes with different bone densities allows the determination of the CT values, and, more interestingly, their change with kV for all densities of cancellous bone encountered, as well as for bone tissue exceeding the density of the total cortical bone samples described above. Figure 2a shows CT values of the bone regions corresponding to extremely soft to very hard bone together with their energy dependence from segmented bone density regions in the porcine tibial head, the bovine ribs, and vertebral bodies from the oxtail samples.

### Soft and adipose tissues

HU measurements of soft (diamonds) and adipose tissues (circles) sampled in the bovine and porcine specimen are shown in Fig. 2b. In soft tissues, no deviation from a flat energy response is found as anticipated. However, samples with highest HU tendentially exhibit a slightly increasing CT value at the soft energy end at 70 kV, while those with lowest CT numbers show a slight reduction. This indicates possible partial volume effects. The adipose tissue samples comprising subcutaneous and visceral fat exhibit average CT numbers between − 39 and − 89 HU at 120 kV with a strong decrease in HU towards softer spectra in accordance with the lower effective atomic number of adipose tissues compared to water.

### Modelling the energy dependence of the tissues

Using all available data from whole bone, cortical bone, and bone segmented in regions with different hardness, a mathematical description the dependence of the CT value on the tube potential for bone tissues was derived. The underlaying idea is, that a single measurement of the CT value at a reference tube potential, in this case 120 kV, is used to calculate the CT numbers of the respective bone tissue at all other kV values from 70 to 140: the CT value as a function of the kV, HU(kV), is modelled based on the CT value measured at a reference kV value of 120 kV (HU(120)) as

1 $${\text{Bone}}:{\text{ HU}}\left( {{\text{kV}}} \right) = {\text{k}}\left( {{\text{kV}}} \right)*{\text{HU}}\left( {{12}0} \right)$$

with k(kV) representing a fourth order polynomial function of the tube potential defined as

2 $${\text{k}}\left( {{\text{kV}}} \right) = {\text{a}}*{\text{kV}}^{{4}} + {\text{b}}*{\text{kV}}^{{3}} + {\text{c}}*{\text{kV}}^{{2}} + {\text{d}}*{\text{kV}} + {\text{e}}.$$

The parameters a to e were determined from the measured data points shown in Figs. 1a, b, and 2a using generalized reduced gradient non-linear fitting minimizing the sum of the residuals^32^. The numerical values of the parameters are provided in Table 1. By using the appropriate ratios of the k values derived, any reference kV value can be used.

**Table 1:** Parameter values of model equation derived from HU measurements for bone and adipose tissues, respectively.

a	b	c	d	e
1.54392E−08	− 8.15985E−06	1.63309E−03	− 1.50596E−01	6.45

α	β	γ		
− 6.80240E−03	1.9698	− 138.43		

In the Figs. 1 and 2a the solid lines were calculated with the model equation using the parameter set from Table 1. Points shown as open circles in the figures correspond to the measured data.

### Soft and adipose tissue

For soft tissue, no energy dependence of the CT values was found, compatible with using a constant CT number independent of scan spectra in the energy range under investigation (Fig. 2b):

3 $${\text{Soft tissue}}:{\text{ HU}}\left( {{\text{kV}}} \right) = {\text{const}}.$$

corresponding to the expected radiologic water equivalency of the tissue mass energy attenuation. Positive CT values in the range of 50 to slightly above 80 HU, thus, correspond to a mass density of approximately 5–8 per cent higher than water. For adipose tissues, due to the lower effective atomic number, CT numbers increase with spectral energy. This increase is best modelled using a kV dependent difference function ∆ to the CT number at a reference kV value of 120 kV,

4 $${\text{Adipose Tissue}}:{\text{ HU}}\left( {{\text{kV}}} \right) = {\text{HU}}\left( {{12}0} \right) \, + {\Delta}({\text{kV}})$$

with

5 $$\Delta \left( {{\text{kV}}} \right) = \alpha *{\text{kV}}^{{\text{2}}} + \beta *{\text{kV}} + \gamma .$$

Similar to modelling the CT number dependence of hard tissues, the parameters α to γ were determined with generalized reduced gradient non-linear fitting from the measured data from the adipose tissue samples shown in Fig. 2b. The resulting parameter values are provided in Table 1. By using the differences in ∆ to any kV value, this value can be used as reference. In Fig. 2b the lower set of curves corresponds to adipose tissue. The solid lines refer to the calculated energy dependence according to Eq. (4), measured data points are shown as circles.

### Comparison of energy dependence of common printing materials with tissues

**Figure 3:**
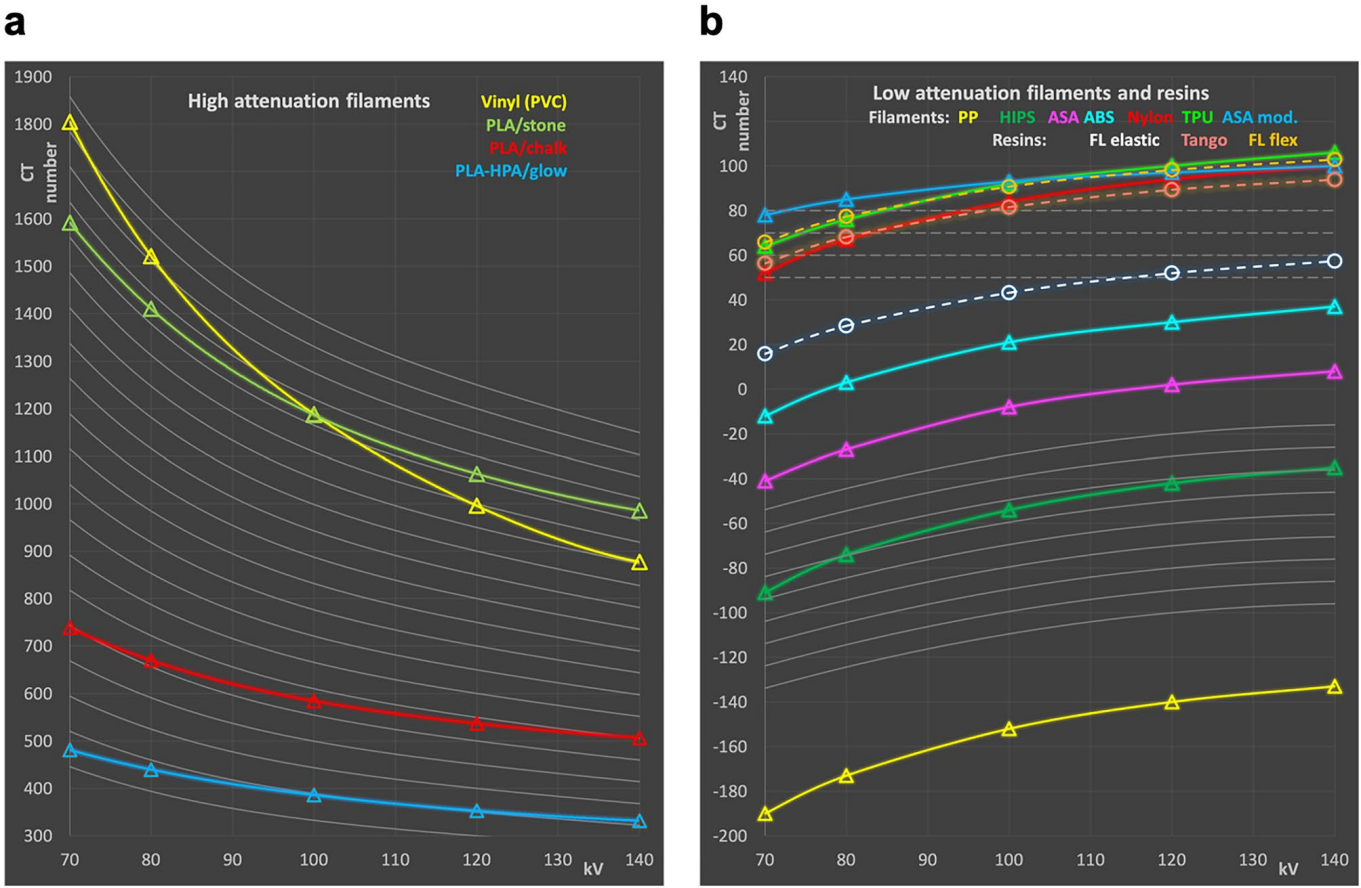
Comparison of 3D printing materials with hard and soft tissues. (**a**) Bone tissue and high attenuation printing materials, and (**b**) soft and adipose tissue and low attenuation filaments. Gray set of curves corresponds to bone tissues with various densities for comparison according to Eq. (1) in panel (**a**), soft (broken gray lines) and adipose tissue (solid gray lines) according Eqs. (3) and (4) respectively, in panel (**b**). Circles connected with broken lines correspond to photocurable resins in panel (**b**) to easily distinguish printing resins from FDM filaments shown as triangles connected by solid lines.

In Fig. 3, CT numbers measured for suitable additive manufacturing materials are compared to the respective values of the tissues using the model equations. 3D printing materials include FDM filaments and photocurable resins.

In the attenuation range of bone tissues (Fig. 3a), four materials have been identified, two of which (PLA/stone and PLA/chalk) contain calcium carbonate powder from stone (most likely limestone or marble) and chalk, respectively, suggesting appropriate energy dependence of attenuation similar to bone tissues. The other two high attenuation filaments were vinyl (PVC), and a material based on PLA/PHA filled with phosphorescent pigment (PLA-HPA/glow). No photocurable resins with appropriate X-ray attenuation capable of simulating bone in CT were available. PLA/stone (shown in green) exhibits the most appropriate energy dependence and seems best suited to mimic denser bone. With a CT value of 1063 HU at 120 kV, it is found in between total bone and compact bone. Compared to the bone model according to Eq. (1), differences in the energy dependence to real bone are minute. This is also illustrated in Table 2 showing HU numbers of the most appropriate 3D printing materials at 120 kV, and differences in HU to the model tissue with the same CT number at this reverence tube potential. Maximum deviation is found at 80 kV with + 15 HU. In PLA/chalk (red in Fig. 3a), deviations from bone tissue at lower energies are larger due to the higher content of the base polymer resulting in a too low effective atomic number. With a CT value of 537 at 120 HU, it corresponds to low density whole bone; however, the maximum deviation to real bone with this density is − 59 HU at 70 kV.

**Table 2:** CT numbers of common printing materials best simulating bone and adipose tissue, and differences to tissue with equal CT value at 120 kV.

	HU at 120 kV	Tissue substituted	∆HU 70 kV	∆HU 80 kV	∆HU 100 kV	∆HU 140 kV
Vinyl	996	Bone	324	214	84	− 38
PLA/stone	1063	Bone	12	15	8	8
PLA/chalk	537	Bone	− 59	− 35	− 11	13
PLA-HPA/Glow	353	Bone	− 43	− 23	− 6	8
HIPS	− 42	Fat	− 15	− 8	− 3	3

Note: value at 120 kV is not provided, since zero.

The same holds true for the phosphorescent filament PLA-HPA/glow. Vinyl exhibits the largest deviation from bone in the energy dependence with − 38 to + 324 HU relative to 120 kV, disqualifying it as bone substitute in X-ray phantoms in case X-ray spectra are varied.

In the range of soft and adipose tissues, seven FDM printing filaments with appropriate attenuation, i.e., above water and less or equal to 100 HU at 120 kV, and three photo curable resins were available. These resins are all elastic or flexible materials, since all rigid resins exhibited too high X-ray attenuation (> 100 HU at 120 kV) and, thus, were excluded from the comparison with soft and adipose tissues.

No off-the-shelf printing material can appropriately mimic the attenuation and flat energy dependence of average soft tissue over the full energy range applied in CT scanning. Due to the too low effective atomic number of all low-density printing materials available, the CT values decrease towards lower spectral energies too strongly.

However, this effect is also evident in fat tissue, since adipose tissue does not only have a lower density than water or other soft tissues like organ parenchyma and muscle, but also a lower effective atomic number. HIPS exhibits the best choice of a material for mimicking CT numbers of adipose tissue in applications where the energy dependence is important, and printing is performed with standard protocols aiming at producing solid printouts with a mass density close to the filament density. It has a relative difference of + 3 HU to − 15 HU to adipose tissue at the highest and lowest energy spectrum investigated, respectively, with identical attenuation at 120 kV used as reference (cf. Table 2).

## Discussion

An important potential use of phantoms is optimization of imaging paraments, and verification of the optimization of clinical protocols. This is especially important in CT, since CT scans are associated with relatively high radiation doses to patients^33,34^. Since results from simple homogeneous phantoms do in most cases not relate to either image quality or dose level estimations in patients^14,35^, it is important that phantoms with realistic structures are used. Optimization of imaging spectra, e.g. by adding additional beam hardening filters like tin filters^36,37^, and automatic or manual variation of tube potential^38^ are becoming standard procedures. If different beam qualities are compared, it is essential that the radiographic contrasts in the phantom exhibits the same photon energy dependence as the tissues it represents. In bone, the CT value changes by over 400 HU in whole/total bone, and approximately 900 HU in cortical bone depending on the tube potential. For adipose tissues the difference with the energy seen in the measurements is up to a little more than 40 HU. With the data on the expected energy dependence of bone, soft and adipose tissues, phantom materials can be benchmarked against these values and selected accordingly. However, no off-the-shelf materials mimicking tissues perfectly are available. With the data presented phantom contrasts can be corrected to real “tissue contrasts” to still define suitable figures of merit, even if kV are varied.

Many approaches have been reported to alter the X-ray interaction properties of common printing materials. To adjust linear X-ray attenuation downwards in FDM printed materials, controlled underfilling reduces the density while maintaining the mass attenuation coefficients of the base material. Controlled reduction of density can be realized in two different ways, either reducing the extrusion rate of the material by controlling the transport speed of the filament in the extruder^39,40^, or by printing dedicated infill patterns with reduced infill^41,42^. The former has the advantage, that macroscopic inhomogeneities can better be minimized or at least kept below the spatial resolution of the imaging system. The disadvantage, on the other hand, is that the underfilling ratio is more limited than when printing infill patterns, which result in visible inhomogeneities especially for low infill ratios.

Reducing the physical density of a material with a mass attenuation coefficient μ/ρ from ρ to ρ’ results in a reduction of the CT value according to

6 $${\text{HU}}(\rho ^{\prime}){\text{ }} = ~\frac{{\rho ^{\prime}}}{\rho }({\text{HU}}(\rho ) + {\text{1}}000){-}{\text{1}}000.$$

**Figure 4:**
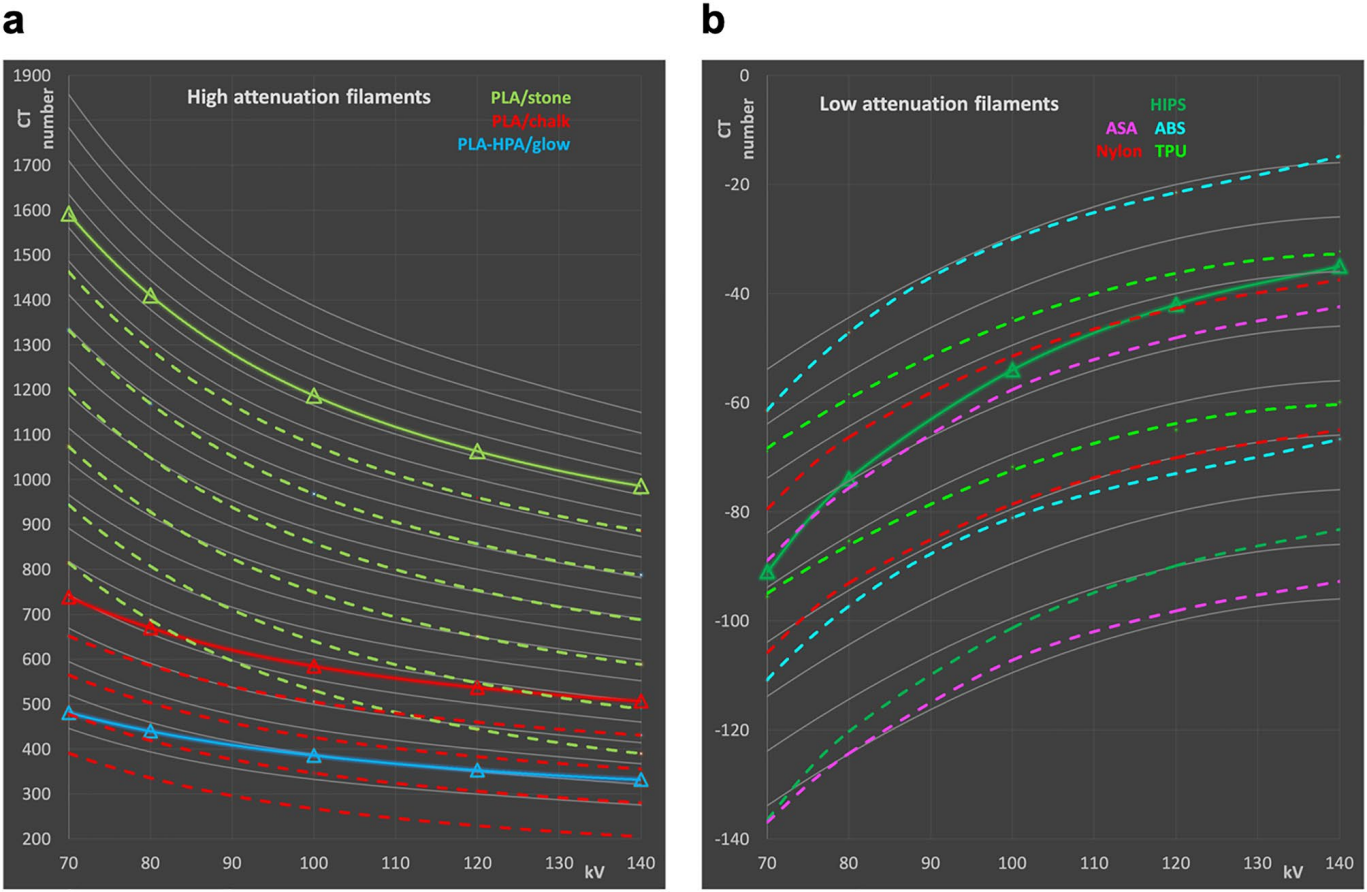
Calculated CT numbers of samples with reduced density (broken lines) compared to bone (**a**) and adipose tissue (**b**) in the HU ranges of interest. (**a**) PLA/stone measured and with reduced density in 5% steps from 95 to 70%; PLA/chalk: measured CT values, and 95–80%, and measured values for PLA/glow. (**b**) HIPS (100%: measured values, and 95%), ASA (95%, 90%), ABS (95%, 90%), Nylon (87.5% and 85%), and TPU (87.5 and 85%), respectively.

Two readily available FDM printing filaments, PLA/stone and PLA/chalk, exhibit in principle favorable energy dependence to duplicate the attenuation of bone tissues. This is due to their chemical composition including powdered calcium compounds. Figure 4a illustrates the potential use of PLA/stone and PLA/chalk printed with reduced densities to reproduce attenuation and energy dependence of medium hard and soft bone tissue. PLA/chalk, e.g., with a filling ratio of 85–90% exhibits an energy dependence very close to soft bone, and far better than PLA-HPA/glow, e.g., which might be seen as a possible candidate material because of its suitable absolute value of attenuation at a single typical tube potential. The same strategy can also potentially be used for duplicating adipose tissues in CT phantoms. In Fig. 4b, especially Nylon and TPU printed with approximately 85–87.5% of its maximum density reproduce attenuation of adipose tissue well, and exhibit a more accurate dependence on the tube potential than HIPS, the only material found in the HU range of typical adipose tissue when printed with 100% filament density. Underfilled vinyl (mimicking bone) and ASA/mod (duplicating fat) are not shown in Fig. 4, since their energy dependence is very different to the target tissues, disqualifying them as appropriate surrogates in phantoms.

This idea could be applied to define “typical” bone and fat tissue substitutes. However, “typical” tissues need to be defined first. Using a standard scan protocol with 120 kV, CT numbers in whole/total bone were found between 458 and 1473. In bones consisting of cortical and spongious bone, like rib bone, vertebrae, fibular and tibial head, the average CT numbers including all bone densities present in the samples ranged from 458 to 624. In hard bones (tibial and fibular shaft), 1116–1473 HU were measured. The highest density bone segments were found in the porcine tibial head. There, the highest density segment which consists of voxels with a CT value above 1950 HU was measured with an average CT value of 2017. Typical human cancellous bone is found at approximately 300–400 HU^43^, while literature values for “typical cortical bone” suggest 500–1900 HU^44,45^. Thus, values measured in the porcine and bovine samples compare to human bone attenuation and provide an appropriate bone model. However, the lower boundary for cortical bone strongly depends on thresholds used for segmentation of cancellous from cortical bone. All CT numbers refer to 120 kV typically used in patient scans.

In phantoms, if no CT values from patient scans representing the anatomy to be duplicated are available, it seems a prudent choice to use a value of 300 to 400 HU for soft bone, 450–650 for average total (whole) bone, and 1000–1500 for dense/compact bone.

In the following, 350 HU are used for a material mimicking soft bone, and 500 HU for average whole/total bone. However, printing compact bone surrogate materials is limited by the densest available filament containing calcium carbonate. PLA/stone, printed with maximum density, results in 1063 HU, thus representing the best choice to mimic cortical bone. Another issue to consider besides attenuation is the energy dependence of the CT numbers. While PLA/stone, owned to the relatively high calcium content, exhibits a very similar energy dependence as hard bone tissue with approximately 1000 HU as illustrated in Figs. 3a and 4a, this advantage is lost when mimicking bone of lesser densities by using controlled density reduction of the printed material. In real bone, the effective atomic number decreases due to a higher bone marrow content for lesser dense bone, resulting in a weaker HU increase for softer spectra (see Figs. 3a and 4a). Figure 4a illustrates how the two calcium carbonate filled printing filaments, when printed with maximum and reduced density, would compare to real bone of different densities, and the potential bone candidate materials printed with their specified density.

The same strategy can also be used for mimicking adipose tissues. To define a single phantom material to duplicate X-ray attenuation and CT numbers of adipose tissue, the following approach may be considered. Voxels containing adipose tissue including subcutaneous and visceral fat in humans typically vary in CT scans to a great extent. If partial volume effects at the tissue boundaries are excluded, a HU window of − 30 to − 140 is reported to best segment adipose tissue volumes from other tissues^46^. The average CT value, then, is quite well defined. By some authors it is reported as − 90 to slightly less than − 100 HU^46,47^ in patients in the abdominal region. Other authors report average adipose tissue values between − 63 to − 88 with an average of − 79 HU for visceral fat, and − 64 to − 104 (average − 89) for subcutaneous adipose tissue^48^ using standard scan protocols applying a tube potential of 120 kV. CT numbers of adipose tissues in this work were higher, with the lowest values seen in the visceral fat segmented in the bovine rib sample. However, samples scanned in this work were smaller in size as compared to human patients. Best accordance is found in the bovine rib sample, with adipose tissue measured at − 61 to − 89 HU. Given the CT values measured, and reported from human patients, a HU range of − 60 to − 100 seems appropriate to imitate fat. If a single value is sought, − 80 HU seems an appropriate choice for a general fat mimicking material at 120 kV. However, none of the printing materials when printed according to specifications falls in this range. Two materials come closest. Polypropylene, however, exhibits a too low attenuation (− 140 at 120 kV), and high impact polystyrene (HIPS) a too high CT value (− 42, also at 120 kV). Using a polymer with a slightly too high density and applying controlled density reduction in the printing process might therefore be beneficial.

Figure 4b shows a comparison of low attenuation filaments including ASA, ABS, Nylon and TPU with reduced density, and HIPS compared to adipose tissue to illustrate the energy dependencies of the CT value for these materials. ASA/mod and all photo cured resins are omitted in the figure, because ASA/mod exhibits a too flat energy dependence to be a candidate for mimicking adipose tissues even when printed with reduced density, and density reduction for photo cured resins printed with SLA, DLP or polyjet printers is hardly feasible.

**Figure 5:**
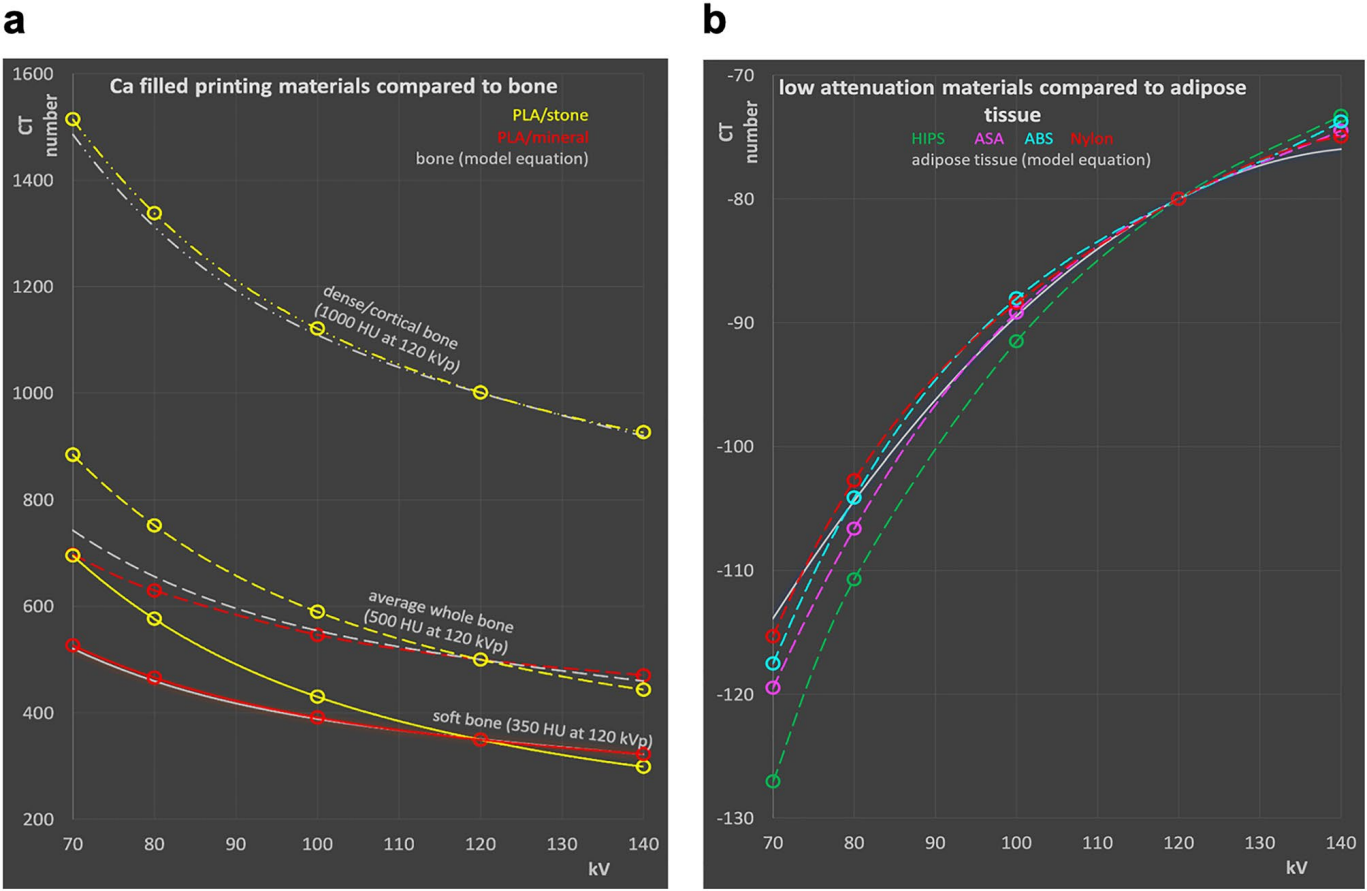
Calculated CT numbers of 3D printing materials best resembling bone and soft tissue. (**a**) Bone tissues: dense/compact bone with 1000 HU at 120 kV mimicked by PLA/stone; average whole bone (500 HU at 120 kV) duplicated by PLA/stone PLA/mineral; and soft bone (350 HU at 120 kV) surrogates made with PLA/stone and PLA mineral, respectively. (**b**) Average adipose tissue with − 80 HU at 120 kV, and printing materials with the same X-ray attenuation at this reference spectrum: HIPS, ASA, and Nylon. All printing materials printed with reduced densities resulting in equivalent HU compared to the tissue mimicked at 120 kV.

Filling ratios to exactly duplicate X-ray attenuation of the tissue at a single kV value can easily be calculated allowing the design of materials simulating these “typical tissues”. The resulting filling ratios are provided in Table 3 for mimicking typical soft bone with 350 HU at 120 kV, average whole (total) bone (500 HU), dense/cortical bone (1000 HU), and average adipose tissue (− 80 HU, all values corresponding to 120 kV) with different printing filaments. Figure 5 illustrates the resulting energy dependences accordingly, and compares CT numbers to those obtained for real tissues using model Eqs. (1) and (4) for bone and adipose tissues, respectively. These differences at other kV settings are also listed in Table 3. In phantoms, where contrasts are printed with these materials, these values can be used to correct measured HU contrasts to “real” tissue contrasts, i.e., when calculating figures of merit containing contrast or contrast to noise ratios to account for non-ideal spectral dependencies of these materials.

**Table 3:** Calculated differences in CT values between the tissue mimicked, and the polymers with equal X-ray attenuation at 120 kV.

	HU at 120 kV	Phantom material and filling ratio	∆HU 70 kV	∆HU 80 kV	∆HU 100 kV	∆HU 140 kV
Soft bone	350 HU	PLA/stone; 65.4%	176	118	43	− 22
		PLA/chalk; 87.8%	7	7	4	2
Average whole bone	500 HU	PLA/stone; 72.7%	142	96	35	− 16
		PLA/chalk; 97.6%	− 46	− 27	− 8	11
Dense/cortical bone	1000 HU	PLA/stone; 96.9%	27	24	11	6
Adipose tissue	− 80 HU	Nylon; 84.1%	− 1	2	1	1
		ABS; 89.3%	− 4	0	1	2
		ASA; 91.8%	− 6	− 2	0	1
		HIPS; 96.0%	− 13	− 6	− 2	3

Dense bone is best duplicated by PLA/chalk, while dense/cortical bone can be appropriately mimicked by PLA/stone. Nylon best imitates adipose tissue, while ABS and ASA are also within a few HU at all energies.

Applying controlled density reduction can be applied to a number of materials. Thermoplastic polyurethane (TPU), is per se difficult to print and not advised to be used with underfilling. The attenuation does not show a more favorable dependence on the spectral form if compared to nylon, e.g., therefore it was avoided in the above discussion.

In this work, mathematical model equations allowing simple calculation of the CT values at all kV values, based on the value measured at a single tube potential, were determined for bone of various densities, and adipose tissues. This allows a comparison and modelling of the energy dependence of bone tissue with different densities, and adipose tissues with various CT numbers. These model equations can easily be used to either compare printing materials with the respective tissues, or to correct CT values in phantom measurements at different tube potentials for a non-perfect energy dependence of the phantom materials, e.g., in contrast and contrast to noise ratio (CNR) based optimization studies. However, it needs to be emphasized, that actual CT numbers depend on spectra used by the scanner. Therefore, scan modes applying differing spectra by, e.g., adding tin filters, will exhibit deviations in both, CT numbers, and energy dependence. In this case, coefficients and model equations need to be adapted.

Achieved mass densities, and thus CT numbers, of materials printed with FDM printers are influenced by a number of factors. These include printing conditions and protocols^21^, inaccuracies and variations in filament diameter, production batch, print job, and chemical variations in materials used in filament production^31^. If printed phantom parts are emersed in a water filled phantom, water absorption of the polymers should also be considered. While minimal in PMMA (0.3%), photo cured printing polymers can take up 1.1–1.5% water, while in others like Nylon up to 8.5% of water can be absorbed leading to swelling and thus changes in mass density^8^. Therefore, it is advised to measure exact X-ray attenuation properties of the materials to be used in an appropriate test setup. While relative energy dependence of the materials will only differ from the curves presented in this work if the filament composition differs, the necessary underfilling ratios or material attenuation might be slightly different due to deviations in the printing process. Alternatively, achieved densities can be measured from printed samples and compared to the densities of the materials used in this work^21^. Thus, differences in achieved densities due to the printing process can be compensated.

## Methods

Bovine and porcine tissue samples from fresh meat samples intended for human consumption containing soft tissue and bones were scanned in a CT scanner. These samples comprised of one porcine knuckle, oxtail, and a bovine rib cut. For scanning the samples were shrink-wrapped twice and treated with disinfectant externally. Bone samples evaluated in the porcine knuckle were fibula, tibia, and fibular and tibial head. Data for bovine vertebral bodies and bovine ribs were derived from 5 samples each, corresponding to 5 individual vertebral bodies from the oxtail, and 5 pieces from individual ribs, respectively. Scanning was performed in a Siemens Somatom Definition AS (Siemens Healthineers, Erlangen, Germany) with 70, 80, 100, 120 and 140 kV applying a standard head protocol with modifications regarding pitch, mA and kV. Scan and reconstruction parameters were the following: standard filtered back projection using a medium soft kernel (H40s), 32 times 0.6 mm collimation reconstructed as 1 mm slices, 1 s rotation time, and a helical pitch factor of 0.6 to allow higher effective mAs for lower image noise. mAs were set to their maximum value for the lowest kV settings (70 kV: 900 mAs, 80 kV: 1100 mAs) resulting in CTDI_vol_ values of 31 and 60 mGy, respectively, in the 16 cm head phantom. For 100–140 kV mAs values were adjusted to result in a CTDI as close to 100 mGy as possible.

**Figure 6:**
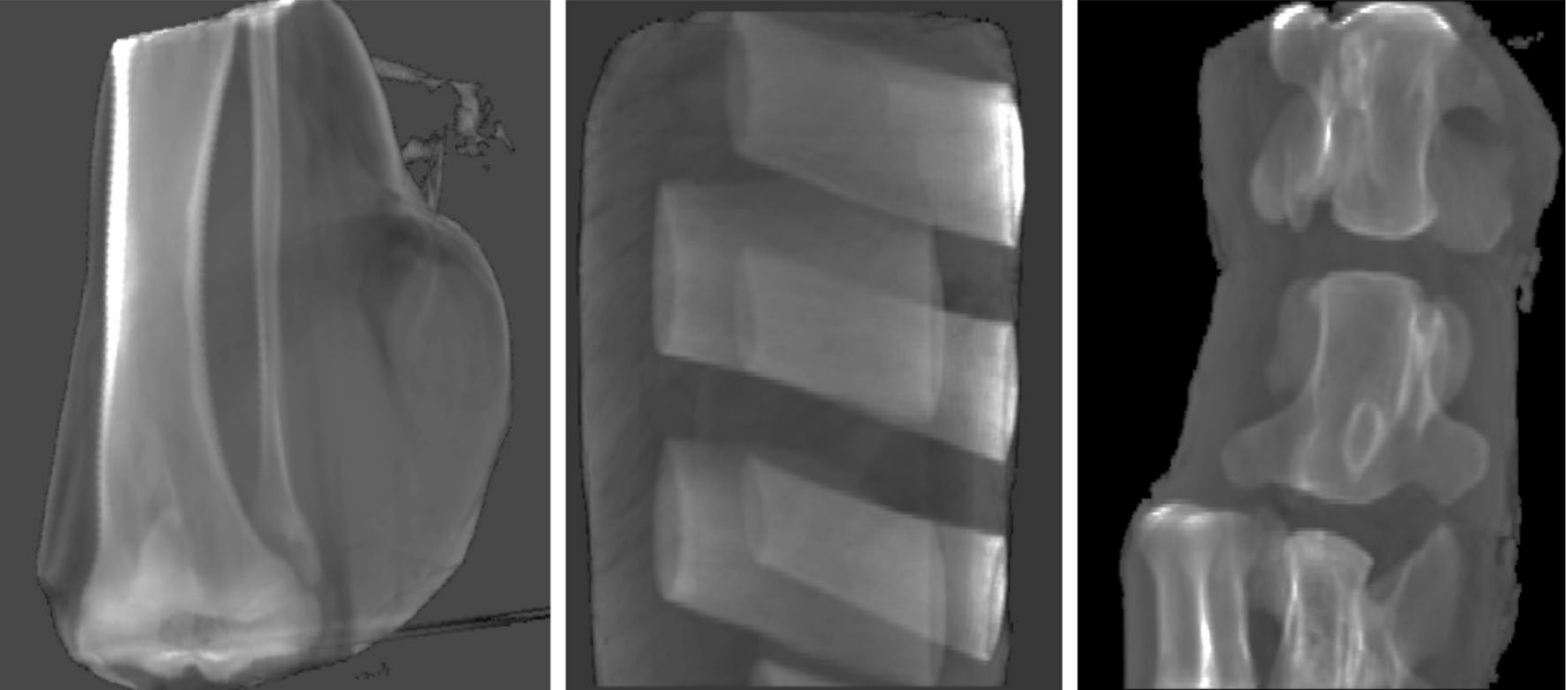
Renderings of porcine and bovine tissue samples applying summed voxel projection. From left to right: porcine knuckle (leg), bovine rib cut, and bovine vertebrae in oxtail sample.

Bone tissue analyzed in this work were the tibial and fibular head and shaft from the porcine knuckle, bovine vertebrae from the oxtail, and rib bone from the bovine rib cut. Soft and adipose tissues were segmented from all samples. Figure 6 shows renderings of the specimen used.

In bone, CT values were determined for whole bone (total bone), cortical bone, and bone segments corresponding to various bone densities from very soft to extremely hard, to derive HU numbers and their energy dependence for a wide variety of bone tissues and densities. Total bone was included to represent average bone as a single material not differentiating cortical from spongious bone segments as used by simpler phantoms. To determine values for total bone, bone has been segmented from the surrounding soft tissues, and yellow bone marrow if present. All segmentation operations were performed on the 120 kV volumes, and also transferred to the registered volumes scanned at the other tube potentials. Registration was performed with subpixel accuracy applying a normalized mutual information algorithm. The outer bone surfaces in the oxtail sample, i.e., the outer surface of the vertebrae, were segmented with a threshold of 300 HU. The same value was used in the bovine knuckle to roughly segment tibial and fibular head, and the outer surfaces of the bone shafts. However, especially in the tibial and fibular heads, the segmentation was corrected manually since thresholding resulted in poor segmentation and manual contouring was required in some slices. Yellow marrow in bone shafts was segmented applying a 50% threshold corresponding to the average value of the cortical bone and bone marrow CT numbers, which was determined with 689 HU. For the evaluation of the cortical bone in the bovine ribs, a segmentation threshold of 900 HU was applied in the definition of the cortical compartment. Removal of the spinal cord in the bovine vertebrae was performed by manual segmentation assisted by thresholding (300 HU).

**Figure 7:**
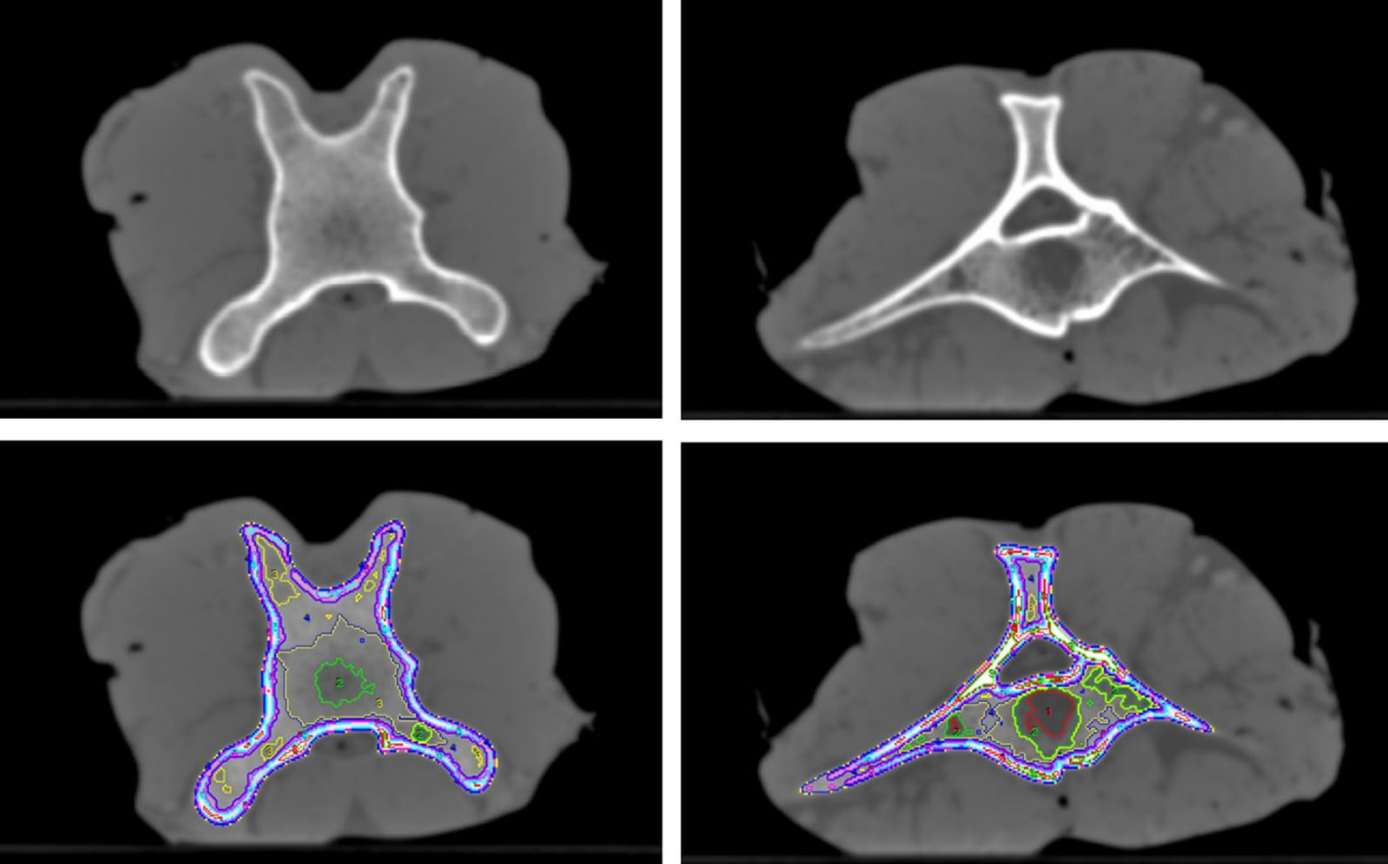
Segmentation of areas with different bone densities using thresholding on the 120 kV image volumes. Example shows two slices from bovine vertebral bodies in the oxtail sample. Upper row: CT image, lower row: CT slice with regions of interest defining the bone density segments.

Bone segments of various bone densities (evaluation shown in Fig. 2a) were delimited by thresholding to determine the energy dependence of bone tissues in wide range of bone densities. This allows appropriate selection of data and thus phantom materials for differently hard (mostly spongious) bone found in different areas of the body. The bone tissue was segmented in density intervals with a width of 200 HU starting at -50 HU corresponding to a central value of 50 HU in the 120 kV volume. Thus, the softest bone measured corresponds to voxels within bone tissue with 50 ± 100 HU, the densest one to CT numbers exceeding 1850 ± 100 HU at the reference tube potential of 120 kV. However, in the porcine tibial head even denser bone was found that could be evaluated allowing to define an extremely dense bone section with above 1950 HU (mean value at 120 kV: 2017 HU). In the bovine ribs bone tissue from 250 HU (central value) to > 1550 HU was present, in the bovine vertebrae from 50 to > 1350, and in the porcine tibial head from 50 to > 1950 HU. Figure 7 illustrates the segmentation showing two slices of two different bovine vertebral bodies from the oxtail sample as an example. Segmentation and the definition of ROIs for HU evaluation was always performed in the scan volume acquired with 120 kV, and transferred to all other scans of the same sample, i.e., those acquired with the other tube potentials, after registration to ensure exactly equivalent ROI placement.

CT values representing adipose tissue were measured in subcutaneous, inter- and intramuscular fat tissue ROIs in the bovine rib cut and the oxtail scans. Inter- and intramuscular fat were segmented from muscle tissue using an upper threshold of − 20 HU. In the porcine leg, only intermuscular and subcutaneous adipose tissue was used for measurements. Average soft tissue was measured in muscular tissue in all samples.

CT numbers were determined for 35 commonly used additive manufacturing materials employing 3D printing, including thermoplastic polymer filaments and photo cured printing resins in a preceding study^21^. Before scanning in a water filled phantom to ensure correct HU numbers and proper function of the scanner’s beam hardening correction, printing parameters of all thermoplastic filament materials were optimized to ensure a maximum printing density to avoid underestimation of X-ray attenuation due to lower than maximum printout density. Scans were performed from 70 to 140 kV, and all data on HU reported. CT scan protocols in the preceding study were identical to the protocols used for the tissue samples in this work. In Table 4 the specifications of the additive manufacturing materials referred to in this work are provided.

**Table 4:** Printing materials used.

FDM materials		
	Polymer description	Filament name and manufacturer
ABS	Acrylonitrile butadiene styrene	ABS transparent; Verbatim GmbH, Eschborn, Germany
ASA	Acrylonitrile styrene acrylate	ASA Extrafill natural; Fillamentum Manufacturing Czech s.r.o., Hulín, Czech Republic
HIPS	High impact polystyrene	HIPS wonderous white, ICE Filaments, Ham, Belgium
Nylon	Polyamide	Nylon transparent; Ultimaker BV, Utrecht, The Netherlands
PLA/chalk	Polylactic acid (PLA) with chalk powder	PLA Mineral natural; Fiberlogy SA, Brzezie, Poland
PLA/stone	PLA with 50% powdered stone	StoneFil Pottery Clay; Formfutura BV, Nijmegen, The Netherlands
PLA-PHA/glow	PLA/Polyhydroxyalkanoate (PHA) with phosphorescent pigment	GlowFill; colorFabb BV, Belfeld, The Netherlands
PP	Polypropylene	Verbatim GmbH, Eschborn, Germany
TPU	Thermoplastic polyurethane	TPU transparent; Extrudr FD3D GmbH, Lauterach, Austria
Vinyl	Polyvinyl chloride	Vinyl 303 natural; Fillamentum Manufacturing Czech s.r.o., Hulín, Czech Republic

Photocured resins		
	Printing system	Resin name and manufacturer
FL elastic	Formlabs Form 2	Formlabs Elastic Resin; Formlabs Inc., Somerville, USA
FL flex	Formlabs Form 2	Formlabs Flexible Resin; Formlabs Inc., Somerville, USA
Tango	Stratasys Objet 500	Tango Plus (translucent); Stratasys Ltd., Eden Prairie, USA

All FDM filaments were printed with an Ultimaker 2, Ultimaker BV, Utrecht, The Netherlands. Photocured resins, since manufacturer proprietary, were printing with the corresponding hardware shown.

All image processing was performed using AnalyzeAVW^49,50^ (Biomedical Image Resource, Rochester, USA). Data analysis was carried out in Microsoft Excel 16.16 (Microsoft Corp., Redmond, USA).

### Ethics declaration and compliance

Methods applied in this work were carried out in accordance of guidelines and regulations. No human and laboratory animal data were used. Ethics committee decision was therefore not necessary.

## Data Availability

The datasets generated during and analyzed in the current study are available from the corresponding author on reasonable request.
